# Lyme Disease and Post-treatment Lyme Disease Syndrome: Current and Developing Treatment Options

**DOI:** 10.7759/cureus.43112

**Published:** 2023-08-08

**Authors:** Norris C Talbot, Noah J Spillers, Patrick Luther, Chelsi Flanagan, Lenise G Soileau, Shahab Ahmadzadeh, Omar Viswanath, Giustino Varrassi, Sahar Shekoohi, Elyse M Cornett, Adam M Kaye, Alan D Kaye

**Affiliations:** 1 Radiology, Louisiana State University Health Sciences Center, Shreveport, USA; 2 Anesthesiology, Louisiana State University Health Sciences Center, Shreveport, USA; 3 Anesthesiology, University of the Incarnate Word School of Osteopathic Medicine, San Antonio, USA; 4 Cellular Biology and Anatomy, Louisiana State University Health Sciences Center, Shreveport, USA; 5 Pain Management, Valley Pain Consultants, Phoenix, USA; 6 Pain Medicine, Paolo Procacci Foundation, Rome, ITA; 7 Pharmacy Practice, Thomas J. Long School of Pharmacy and Health Sciences University of the Pacific, Stockton, USA

**Keywords:** post-treatment lyme disease syndrome, treatment, azlocillin, erythema migrans, doxycycline, ptlds, lyme disease

## Abstract

Lyme disease and its treatment implications have become an ever-increasing area of concern within the United States related to the markedly increased prevalence of infection within the last two decades. The presentation, pathophysiology, and epidemiology of Lyme disease have been well studied, and thus treatments for this disease are widely available. While the treatment of its early and late stages is relatively simple with 10-14 day and four-week courses of doxycycline, respectively, the main problem rests in the understanding of the etiology and pathology of post-treatment Lyme disease syndrome (PTLDS). With the time of symptoms onsetting approximately six months after treatment and potentially lasting indefinitely, this syndrome's effect on patients' quality of life could be devastating. Searching on PubMed, Google Scholar, MEDLINE, and ScienceDirect using keywords including Lyme disease, PTLDS, doxycycline, erythema migrans, azlocillin, and treatment, the authors have tried to make clear the different aspects. The authors have reviewed and discussed clinical studies of Lyme disease and its treatments/potential therapeutics as well as PTLDS and its sparse treatments/potential therapeutics.

## Introduction and background

Lyme disease is one of the most prevalent vector-borne diseases in the United States [1]. Originating from the spirochete family of bacteria, specifically the genus *Borrelia*, Lyme disease can be caused by different strains depending on geographical location. *Borrelia burgdorferi* is found in the western hemisphere, and *B. afzelii* and *B. garinii*, in addition to *B. burgdorferi*, can be found in Europe and Asia [2]. *B. burgdorferi* often causes arthritic symptoms while *B. afzelii *and *B. garinii* commonly cause skin and neurological manifestations [3]. Commonly, the cases of Lyme disease in the United States have increased in the northeastern portion of the country as well as the mid-Atlantic and upper Midwest [4]. European cases are seen in the Scandinavian and Baltic states, in Northern Europe, Germany, Austria, Czech Republic, and Slovenia in Central Europe [4]. Furthermore, a new species, *B. mayonii*, was discovered as rarely appearing in the upper Midwest and is not known to exist in Europe [4]. The specific vector is primarily the *Ixodes* species of ticks, which acquire the spirochete from smaller animals such as birds and mice by taking a blood meal to molt. From this point, the ticks utilize the blood meal to transition from larva to nymph or nymph to adult tick. If the bacteria survive this developmental stage, they can remain in the nymph or adult tick stage and be transferred in the next blood meal, which can commonly involve deer and humans [1].

Approximately 476,000 people are treated for Lyme disease each year in the United States [5]. Since 2004, Lyme disease has accounted for 63% of all reportable tick, flea, or mosquito-borne illnesses nationwide [1]. The major health concerns of Lyme disease begin with characteristic skin rashes and can eventually lead to dermatological, cardiological, musculoskeletal, and neurological manifestations [6]. Furthermore, the consequences of infection with Lyme disease can lead to continuing symptoms and also develop into post-treatment Lyme disease syndrome (PTLDS). Of Lyme disease patients, 10-20% will not respond to treatment and can develop PTLDS after the application of antibiotics [7]. These patients will continue to experience prolonged somatic and neurocognitive symptoms. While Lyme disease has several clinically approved treatments, PTLDS suffers from the limited options of only therapeutics directed toward symptom management rather than attacking the disease. Recent studies show that PTLDS may be due to the development of an underlying autoimmune condition after treatment with antibiotics [8,9]. Furthermore, reports of Lyme disease have expanded in the last decade, showing marked spread into previously non-endemic areas [1]. This review aims to inform healthcare practitioners of the growing risk of Lyme disease in previously low-incident areas within the United States. We analyze in this review the pathophysiology, epidemiology, presentation, diagnostics, and current and developing treatment for Lyme disease as well as pathogenesis and current treatment for PTLDS.

## Review

Methods

This was a narrative review. The sources for this review are as follows: searching on PubMed, Google Scholar, MEDLINE, and ScienceDirect using keywords including Lyme disease, PTLDS, doxycycline, erythema migrans, azlocillin, and treatment.

Lyme disease pathophysiology/epidemiology/presentation

Pathophysiology

Lyme disease is a vector-borne illness caused by the acquisition of bacteria in the host through a deer tick bite. Primarily found in North America and Europe, the dominant vector is the tick *Ixodes ricinus* [10]. These ticks are well-known to be common carriers of the causative bacterial agent, *Borrelia burgdorferi* [10]. The majority of Lyme disease cases in the United States are found in the Northeast, Mid-Atlantic, and Mid-Western states where there is an abundance of trees, ticks, mice, and deer, contributing to the spread of the disease [2]. When these ticks bite potential carriers such as mice or deer, the spirochetes are transmitted through saliva. Likewise, the ticks will take a blood meal to molt from smaller animals, allowing the bacteria to progress with the tick into its next life cycle as a nymph or adult [11]. Furthermore, the ticks in later life cycles with surviving *Borrelia burgdorferi* can transmit it through their saliva into the next blood meal, which can include humans. Once these bacteria breach the skin's physical barrier, they use the tick’s saliva and surface proteins to avoid the immune system by modulating host activities, including coagulation, fibrinolysis, and the immune response [12]. After initial immune system evasion, these bacteria use their spirochete shapes to disseminate locally among nearby tissues. The majority of bacterial dissemination into deeper tissues from the site of the bite occurs through hematogenous spread [13]. One paper noted that both bacterial and host proteins specifically interact to contribute to pathophysiologic processes [13].

Epidemiology

**Figure 1:**
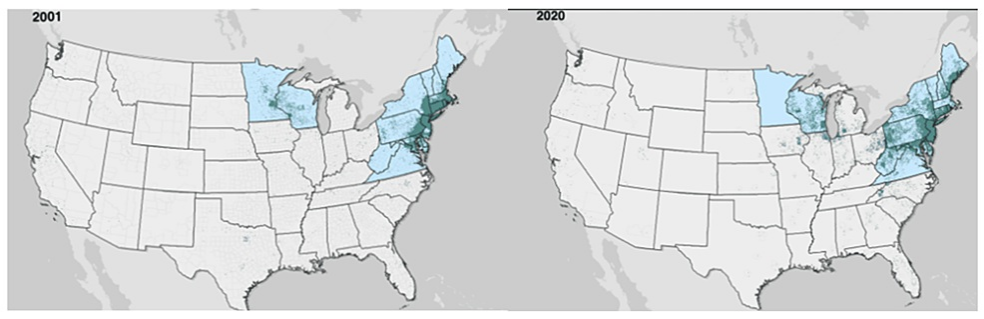
Data sourced from CDC reports of Lyme disease in 2001 vs. 2020. The lack of dots in Massachusetts in 2020 is related to a difference in reporting standards. Figure adapted from [16].

Lyme disease is primarily found in pediatric populations in ages five to 15 years and adult populations aged 45-55 years [1,14]. The disease is also found more often in men than women under the age of 60 years [13]. In populations over the age of 60 years, the prevalence was equal or higher in women compared to men [13]. In a study on the increasing incidence of Lyme disease from 1993 to 2012, the number of high-incidence counties in the United States increased from 69 counties to 260 counties [15]. Upon analysis of CDC-reported data, Lyme disease in the past 20 years has become increasingly more prevalent in the Mid-Western states, but the amount of confirmed cases has remained consistent since 2010 [16]. The incidence rate was 9.6 people per 100,000 in 2012 and 10.6 people per 100,000 in 2019, but it was 5.5 people per 100,000 in 2020 [16]. Notedly, these incidence rates include the entire United States, including states outside of the highly affected regions of the US. The marked decrease in 2020 is attributed mainly to the COVID-19 pandemic since fewer people sought care for tick bites and significantly fewer laboratory tests were ordered [17]. While the total number of cases of reported Lyme disease has generally remained consistent, except in 2020, the data displayed a larger geographical dissemination into states that previously had small amounts of reported cases. The increased incidence of Lyme disease from 1993 to 2012 is largely influenced by human behaviors and changing landscape environments that affect the number of ticks and small mammal species to spread the disease [15]. While the incidence of Lyme disease remarkably increased from 1993 to 2012 and has remained consistent until 2020, the geographical expansion, which had not seen extensive movement from 1993 to 2012, has now increased by 2020 to have higher incidences in previously low-incidence areas. For example, Michigan had an incidence of one person per 100,000 in 2012 and increased to 4.7 people per 100,000 in 2020 [16]. Ohio shifted from 0.6 to 3.5 people per 100,000 in the same time period. While states like Pennsylvania and Rhode Island increased from 39.4 and 17.2 people in 2012 to rates as high as 70.3 and 91.8 people per 100,000 in 2019, respectively [16]. Notedly, both had decreased in 2020 to 26.1 people per 100,000 in Pennsylvania and 79.4 people per 100,000 in Rhode Island. Other states with comparable increases in Lyme disease rates include Indiana, Iowa, Maine, North Dakota, Vermont, and West Virginia, and the District of Columbia also had equivalent rate increases. Other high-rate states such as New Hampshire and New Jersey shifted from 109.5 to 125.7 people per 100,000 and 40.4 to 40.7 people per 100,000, respectively, from 2012 to 2019 [16]. Furthermore, the changes in incidence across a broader geographical area are likely due to the same environmental changes and human behavior that caused increases from 1993 to 2012. The large spread and geographical expansion of Lyme disease prevalence in the past 20 years are exhibited in Figure 1 based on the CDC data.

Presentation

Lyme disease’s clinical manifestations can be divided into three stages. The first stage tends to involve flu-like symptoms and a localized growing rash, referred to as erythema migrans [18]. The second stage, the early disseminated stage, can present with more severe conditions such as carditis, borrelial lymphocytoma, and Lyme neuroborreliosis [18]. In these stages, dissemination is likely to involve the nervous system [18]. Nervous system involvement can present as neck or joint stiffness and/or facial palsy [18]. Bell’s palsy is the most common facial palsy involved in Lyme disease and is found with both unilateral and bilateral involvement [18]. Nervous system radiculopathies have also been found in this stage but usually occur due to untreated Lyme disease [10]. Second-stage Lyme disease can also contain more dermatological findings similar to the primary rash, but the new rash is smaller and also spreads to distant locations [18]. The third stage consists of inflammatory oligoarthritis usually involving asymmetric weight-bearing joints, typically the knee [19]. The symptoms start approximately two months after the initial infection with no treatment and can range from mild to moderate pain to severe excruciating pain with erosive joints [19]. Importantly, the manifestation of Lyme disease symptoms can be sporadic and not follow a predictable order, leading to problems with diagnosis.

Diagnostics

Since the initial emergence of Lyme disease, there has been substantial progress made in proper identification and diagnosis. A positive serological diagnosis can be made in each of the stages of the disease. However, due to different levels of circulating antibodies in patients at separate timelines of the disease, the probability of a positive serological test varies depending on disease progression. Circulating antibodies are generally detectable during the first two stages [20]. The current standard for Lyme disease diagnostic testing is a two-tier serologic test for antibodies to *B. burgdorferi* [20]. These tests usually involve a first step quantitative stage, usually with an enzyme-linked immunosorbent assay (ELISA) [20]. This procedure is promptly followed with a more specific western immunoblot where a positive test only occurs if both the ELISA and western immunoblot are positive [20].

There is some slight variation in what level of antibody binding is considered positive, so it is imperative that labs follow the CDC guidelines on immunoblot interpretation [20]. Specifically, the western blot requires five bands to be positive. The stages of Lyme disease can have significant symptom overlap, which physicians must identify to order the correct diagnostic tests. The typical erythema migrans rash, often referred to as a localized disease, does not always follow the typical pattern or progression. If a patient were to present after the initial rash resolves but still has similar symptoms, some physicians could completely dismiss the possibility of Lyme disease. As a result, this dismissal can cause both overdiagnosing or misdiagnosing patients with post-treatment Lyme disease. For example, if a patient presenting with lethargy, low energy, and low appetite had a history of Lyme disease in the past, there can be an overdiagnosis of post-treatment Lyme disease. Conversely, if a patient previously had undiagnosed Lyme disease and presented with the same symptoms, there could be a misdiagnosis of various mental health issues, including depression [18]. Without appropriate serological testing or in the event of poor interpretation, the combination of the variability of the timeline of symptom presentation and the different array of symptoms that can be involved make this disease a challenging condition to diagnose accurately and subsequently presents increased difficulty in treating [21].

Post-treatment Lyme disease syndrome pathogenesis

One of the most concerning aspects of Lyme disease is the presence of symptoms after treatment. This trait is characteristic of an aptly named post-treatment Lyme disease syndrome (PTLDS). The Infectious Diseases Society of America participated in a panel meeting in 2006 for formal recognition of the ambiguity of the definition of PTLDS and suggested that a future agreed-upon definition should include empirical evidence [22]. Although the exact pathogenesis of PTLDS is still undetermined, a few theories exist to try to explain the occurrence of these symptoms. Controversy over the exact definition of PTLDS stems from little to no evidence supporting a chronic *B. burgdorferi* infection; however, it is possible that there may be active pathology that occurs after the initial clearance of the organism. Since the typical causative agent, *B. burgdorferi*, does not produce exotoxins, one theory suggests that the acute symptoms of PTLDS are due to both the innate and adaptive immune systems working to clear lingering bacteria [20]. The associated symptoms including myalgias, stiffness, fatigue, and other symptoms are caused by the subsequent inflammatory cytokine release from the immune system [20]. Another theory states the chronic aspect of arthritis in PTLDS is due to the slow clearing of peptidoglycan from the joints [20]. These authors also suggest that *B. burgdorferi* and its antigens could remain pathogenic even after adequate antibiotic treatment [20]. This hypothesis suggests that the bacteria's ability to undergo adaptations, prompted by antibiotic-related stress, allows it to enter a persister phase similar to biofilm formation [20]. Another theory states the possibility of autoimmunity of the host immune system producing auto-antibodies to bacteria peptidoglycan, which causes the continuous release of cytokines, which may be the reason behind the chronicity of these symptoms [20]. The final point states the idea of central sensitization, which is increased responsiveness to noxious stimuli even if the stimulus may be normally sub-threshold [20]. These theories still lack the appropriate evidence to elucidate the true pathogenesis of PTLDS, prompting the need for more research into these proposed mechanisms.

Current treatment for early Lyme disease and post-treatment Lyme disease syndrome

Per guidelines from the CDC, erythema migrans rash is treated with doxycycline 100 mg twice per day orally for 10-14 days, amoxicillin 500 mg three times daily for 14 days, or cefuroxime 500 mg twice a day orally for 14 days [23]. These findings resulted from several studies that found high efficacy with treatment for their respective drug courses.

The effectiveness of doxycycline, in Lyme disease treatment, was supported in a study with 607 patients, 93% of which were treated with courses of doxycycline in 11, 11-15, and 16-day regimens. Treatment failure was found in less than 1% of patients, more than half of which were found to have symptoms suggesting reinfection [24]. However, doxycycline antibiotics carry rare potential adverse effects, including photosensitivity, pseudotumor cerebri (PTC), and esophageal perforation [25]. The effectiveness of cefuroxime vs. doxycycline as a treatment in adult erythema migrans infections was evaluated in a study that found satisfactory remission of symptoms in 51 of 55 patients treated with cefuroxime and 45 of 51 patients treated with doxycycline [26]. Another study was performed concerning the effectiveness of cefuroxime as an alternative to doxycycline as a treatment option for children with erythema migrans [27]. This study found total resolution of symptoms in 92% and 67% of groups treated with cefuroxime and doxycycline, respectively [27].

PTLDS has proven to be more difficult to remediate as the specific nature of its pathology and etiology is poorly understood. Many patients experience symptoms that include fatigue, pain, arthralgia, and neurocognitive involvement [28]. There is very little literature referencing specific therapies for symptoms of PTLDS, but in recent times, the topic has been extensively reviewed [29]. Treatments for symptoms of arthralgia and pain are generally done so in a stepwise manner, from heating pads and nonsteroidal anti-inflammatory drugs to physical therapy and narcotics. The treatment for neurocognitive involvement depends heavily on the specificity of the deficits that the patients present with. According to the Mayo Clinic, the cessation of some drugs that may be involved with exacerbating neurocognitive symptoms has been indicated in patients suffering from mild cognitive impairment [30]. Medications that can exacerbate neurocognitive symptoms include drugs such as benzodiazepines, anticholinergics, antihistamines, opioids, and proton pump inhibitors [29]. The length of this syndromic condition appears to have a wide range as one study by Rebman et al. [31] had a cohort of patients whose range of PTLDS onset was from 8.3 months to 27.7 years. For patients on medications for comorbid or chronic conditions, this can present difficulties in their treatment and lead to overall reductions in quality of life.

While potential therapeutics for PTLDS are currently being investigated, several contraindicated therapeutics have been thoroughly reviewed and are recommended to be avoided by institutions such as the CDC. The risk of infection or electrolyte imbalances in patients with PTLDS treated with oral or intravenous (IV) antibiotics was more prevalent than in those not on antibiotic therapy [32]. Another study of the repeated use of IV and oral antibiotics in five patients found an increased risk of infection accompanied by no improvement in symptoms associated with chronic Lyme disease [33]. A case study, in 2017, evaluated the use of ceftriaxone in a patient with PTLDS that caused acute kidney injury and associated hemolytic anemia [34]. The above studies have not seen significant symptom relief upon administration of antibiotics; thus, it is unlikely that the causative agent of PTLDS is a latent infection of *Borrelia burgdorferi*. Due to the lack of a definitive standard of treatment for PTLDS outside of supportive care, these studies are imperative in improving our ability to set a better standard of care [29]. With current treatments for PTLDS being exclusively supportive, new therapies must be developed to curb the unclear course of this syndrome.

Lyme disease potential therapeutics and prevention options

Vaccination Developments

A previous vaccine, LYMErix, was originally on the market from 1998 to 2002 in which the lipoprotein OspA was targeted with antibodies [35,36]. These antibodies would target the spirochete in the midgut of the tick while feeding; therefore, inhibiting transmission. However, the vaccine received scrutiny due to unsubstantiated claims that correlated the vaccine with arthritis [36]. Despite the FDA stating these claims were unsupported by concrete evidence, the vaccine was pulled from the market in 2002 because of decreased sale revenue [36]. Recent research on the OspA vaccine has served to further remove these connections and show its efficacy in preventing Lyme disease [36].

Some methods of antibiotics tend to work as discussed before; however, with 10-20% of people later presenting with PTLDS and the incidence rate steadily increasing, preventative measures and treatments are necessary to prevent long-term effects [7]. While LYMErix was attempted in the early 2000s, the development of new vaccines has been limited, despite increased rates of Lyme disease [37]. There is no Lyme disease vaccine that has full FDA approval and is currently disseminated in the United States [38]. However, recently, a new vaccine, the VLA15 vaccine, has proven efficacious thus far in mice. The target in mind is once again the OspA protein that exists on the outer side of the spirochete before transmission while it is in the tick’s gut [39]. As the tick feeds, the OspA protein will be downregulated as OspC is upregulated and subsequent infection of the host occurs. The new VLA15 vaccine creates anti-OspA-specific antibodies, preventing tick transmission before OspC can be expressed [39]. Proven by earlier data and experimental studies, the vaccine successfully prevented infection with Lyme disease in mice [40]. Moreover, the vaccine showed the potential to work against many clinically relevant strains of Lyme disease, providing protection in the United States, Europe, and potentially worldwide [39]. The vaccine has progressed to a phase 3 clinical trial, starting in 2022, and is one of the most promising Lyme disease vaccine trials in the past 20 years [41].

Furthermore, studies have been conducted in recent years to discuss the willingness of people to receive a safe Lyme disease vaccine. Recent data of 3313 respondents from northeastern parts of the United States, including Connecticut, Maryland, Minnesota, and New York, showed that 64% of participants were willing to receive a potential vaccine. In contrast, 30% were uncertain and 7% were unwilling [42]. Importantly, those who were unwilling tended to be generally dissatisfied with vaccines and were not correlated to a Lyme disease vaccine specifically [42]. Ultimately the VLA15 vaccine shows the potential to work as a clinically useful vaccine and has public support to be utilized.

Pharmacologic Developments

While the development of a new vaccine and current antibiotics for Lyme disease exist, the prevalence of PTLDS has continued to fester without any treatment for the ambiguous pathology of the disease. Due to the limited understanding of the underlying etiology of PTLDS, current and developing treatments for this condition are centered on preventative rather than curative measures. One theory, mentioned earlier, suggests that PTLDS can arise from persistent and drug-tolerant *Borrelia* infection [38]. Promisingly, a recent study showed the efficacy of azlocillin as a potential candidate for the prevention of PTLDS [43]. However, a large dilemma concerning this potential therapeutic is azlocillin’s broad range of action that commonly creates a more rapid resistance profile among the bacteria it treats [44]. Unfortunately, the potential therapeutics for PTLDS are largely based on theory without convincing evidence. For example, persistent bacteria, dormant and undetectable infections, and triggering an auto-immune response are potential theories for the cause of PTLDS [45]. As a result, azlocillin would have the potential to work only if the theory concerning persistent *Borrelia* as the cause of PTLDS is true. Azlocillin’s current trials in mice studies show curative potential and at least a possible step in the right direction for treatment [43].

To answer the problem of wide-ranged antibiotics, a new drug, hygromycin A, has been found to kill spirochetes selectively [46]. While the current data has only been shown in mice, the low-scale production of Lyme disease treatment presents hygromycin A as one of the more promising developments. Furthermore, the benefits of hygromycin A include sparing the microbiome, low level of resistance development, and no detectable cytotoxicity against human cells [46]. Although the drug was capable of clearing *B. burgdorferi* infection, the current trials only saw this result in mice, so any future use in humans will need extensive testing and research. On the other hand, hygromycin A has been shown to work as a bait for mice and could serve as a method to start lowering the levels of Lyme disease in nature [46]. Moreover, a potential cause of PTLDS has been theorized to be a characteristic shift in the microbiome composition, which hygromycin A has been shown to not affect [47]. While the potential results are promising, extensive research and trials are required before use in people.

Clinical Studies of Lyme Disease Treatments

One challenging aspect of treating Lyme disease is the emergence of antimicrobial-resistant *Borrelia burgdorferi* infections. While the exact cause of PTLDS is unknown, current research postulates that antimicrobial-resistant *Borrelia burgdorferi* infections may be integral in its pathophysiology [48,49].

Several in vitro studies have demonstrated that *B. burgdorferi* forms drug-tolerant "persister cells" when treated with traditional antibiotic therapy and infections by these drug-tolerant forms cannot be eradicated by ceftriaxone and doxycycline [50-52]. In an in vitro study using a semisolid plating method, Pothineni et al. [43] demonstrated that when used alone, the antibiotic azlocillin can completely eradicate late log phase and seven to 10 days old stationary *B. burgdorferi*. The combination of azlocillin and cefotaxime can effectively kill doxycycline-tolerant *B. burgdorferi*. Additionally, the authors found that azlocillin has shown significant efficacy in treating *B. burgdorferi* in mice through in vivo testing. The authors concluded that more in-depth research is necessary to evaluate the potential use of azlocillin in treating Lyme disease and its associated disorders.

In 2020, Wormser et al. [53] described the aggregation of data from four clinical studies focusing on single-dose doxycycline as postexposure prophylaxis for three spirochetal infections: Lyme disease, syphilis, and tick-borne relapsing fever. In all the studies, a single dose of doxycycline was administered within 72 hours of participants’ exposure to one of the spirochetal pathogens. The authors detail a double-blind, placebo-controlled trial in which a single 200 mg dose of doxycycline was administered within 72 hours after detaching an *Ixodes scapularis* tick from the skin; this study found that the postexposure doxycycline administration was 87% effective in preventing the development of Lyme disease [53]. Another open-label, randomized clinical trial performed in 2020 administered a single dose of doxycycline to study participants within 72 hours of an *Ixodes ricinus* tick bite; this study found the efficacy rate of doxycycline was 67% [54]. Through analysis using the DerSimonian-Laird method for variance estimation, the four studies in combination demonstrated an overall efficacy of doxycycline use of postexposure prophylaxis for prevention of the three spirochetal infections as 78% [53]. Another focus of Lyme disease treatment has been the early treatment of the disease to prevent the development of PLTDS. In a longitudinal prospective cohort study, the authors set out to determine if study participants with a prior history of Lyme disease were more likely to meet the criteria for PTLDS than those without a history of Lyme disease [55]. Study participants completed surveys to evaluate their pain, fatigue, depression, and quality of life and afterward, the distribution of clinical outcomes was examined. After the study, the authors found that 13.7% of participants with a history of Lyme disease met the criteria for PTLDS as opposed to the 4.1% of participants without a history of Lyme disease who met the criteria for PTLDS. Overall, patients with prior Lyme disease were about 5.28 times more likely to meet PTLDS criteria when compared to the group without a prior history of Lyme disease.

Kundalini yoga has also been examined as a potential treatment for PTLDS; yoga has been shown to alleviate fatigue, pain, sleep disturbance, and cognitive impairment [56]. Additionally, contemplative practices have been theorized to mediate these symptoms due to their effects on the autonomic nervous system and attenuation of the stress response. Stress is a common occurrence in patients suffering from any type of chronic disease [57].

In 2022, Murray et al. [56] conducted a preliminary randomized study to determine the adherence to and potential benefit of Kundalini yoga for PTLDS. A total of 29 participants were randomly assigned to either eight weeks of Kundalini yoga in group sessions or a “waitlist” control group. Participants were invited to participate in the study if they were 18 years of age or older, had received a clinical diagnosis of Lyme disease at least six months before the start of the study, and had a primary complaint of pain or fatigue that met the required severity criteria. The primary outcomes measured were pain, fatigue, and global health; secondary outcomes included symptom burden, cognition, mood, sleep, and mindfulness. Outcomes were measured using group discussion, the Neuro-QoL Cognitive General Concerns questionnaire, and documentation of independent yoga practice each day. After the study ended, there were no differences in primary outcomes between the two groups. Still, Kundalini yoga was associated with improvement in two of the secondary outcomes: symptom burden and cognition. The authors concluded that Kundalini yoga is safe and cost-effective and may effectively decrease some of the symptoms associated with PTLDS [56].

Investigation into vaccines for the prevention of Lyme disease has become more prominent in recent years. LYMErix is a vaccine for Lyme disease that was developed in the 1990s and was found to reduce infections in vaccinated adults by 80%. However, it was discontinued by the manufacturer due to the low use of the vaccine by the general population [56]. Because Lyme disease is challenging to diagnose and is debilitating when not treated promptly, prevention is key in protecting both children and adults from the disease. Kamp et al. [58] designed a six-component vaccine targeting OspA that can elicit antibody response against all *Borrelia* strains that cause Lyme disease. OspA is a lipoprotein located on the outer membrane of *B. burgdorferi*, and antibodies against this lipoprotein can kill the organism in the midgut of the *Ixodes* tick before transmission occurs. The previously available vaccine, LYMErix, also targeted this lipoprotein but was only effective against one strain of *Borrelia*. They designed the vaccine by fusing OspA to the N-terminus of *Helicobacter pylori* ferritin and was tested in a mouse model. *Ixodes* tick-fed mice that received the vaccine demonstrated immunity from both *B. burgdorferi* and the response was sustained for more than six months. The clinical studies related to the treatment of Lyme disease are outlined in Table 1 [59].

**Table 1:** Treatment regimen studies for Lyme disease. PTLDS: post-treatment Lyme disease syndrome.

Author and year	Methods	Results	Conclusions by the study authors	Inference
Pothineni et al. (2020) [43]	In vivo and in vitro studies were performed using a semisolid plating method to determine the efficacy of azlocillin in treating doxycycline-tolerant *Borrelia burgdorferi *infections.	Azlocillin was found to completely kill the late log phase and 7-10 days old stationary phase *B. burgdorferi.* Azlocillin and cefotaxime combination can effectively kill in vitro doxycycline-resistant *B. burgdorferi.*	The authors concluded that further in-depth research is required to fully evaluate the potential use of azlocillin to treat Lyme disease.	Azlocillin has the potential to become a new standardized therapy in the treatment of Lyme disease, but more research is required.
Wormser et al. (2021) [53]	Analysis of four studies evaluating the use of doxycycline as postexposure prophylaxis for prevention of spirochetal infections: Lyme disease, syphilis, and tick-borne relapsing fever.	A single 200 mg dose of doxycycline was 87% effective in preventing the development of Lyme disease.	The authors concluded that a single-dose doxycycline regimen effectively prevents these three spirochetal infections.	Post-exposure prophylaxis using doxycycline is effective and should be considered in patients exposed to Lyme disease.
Aucott et al. (2022) [55]	The longitudinal prospective study included 234 study participants; all participants completed surveys to assess their fatigue, pain, sleep, and quality of life. Afterward, the distribution of clinical outcomes was examined.	13.7% of participants with a history of Lyme disease met the criteria for PTLDS compared to 4.1% of participants without a history of Lyme disease who met the PTLDS criteria.	The authors concluded that study participants who had been previously diagnosed and treated for Lyme disease were more likely to meet the criteria for PTLDS than those who had not.	This study demonstrates that prior history of Lyme disease may be associated with the development of PTLDS, further emphasizing the idea that prevention is key in treating Lyme disease.
Murray et al. (2022) [56]	A preliminary randomized study evaluating the effects of Kundalini yoga (KY) in treating post-treatment Lyme disease syndrome (PTLDS). Twenty-nine study participants were assigned to either KY group sessions or a waitlist control group. Primary outcome measurements evaluated pain, fatigue, and global health. Secondary outcome measurements assessed symptom burden, mood, cognition, and mindfulness.	There was no significant difference between the groups in relation to primary outcomes. However, KY participants reported improved secondary outcomes (symptom burden and cognition) throughout the study.	This preliminary data suggests that KY may effectively reduce some symptoms associated with PTLDS.	This is the first study that examined a behavioral intervention for PTLDS instead of antibiotic therapy.
Kamp et al. (2020) [59]	Tick-fed mice were vaccinated with OspA-ferritin nanoparticles, and their immune response was followed.	Vaccination with the adjuvanted OspA-ferritin nanoparticles demonstrated immunity against *B. burgdorferi *and *B. afzelii* infection. The response persisted for more than six months.	The authors concluded that this vaccine can potentially limit the spread of Lyme disease.	Prevention is key in treating Lyme disease; developing a vaccine that has been effective in a mouse model is promising.

## Conclusions

The growing prevalence and exposure to Lyme disease have created an increased necessity for newer treatments and medicines to combat the rise in cases. Furthermore, since the rise in Lyme disease is paired with difficulty in diagnosis, long-term complications have also increased. As a result, the need for clear and concise treatment options and methods has become more important as well as an emphasis on research for new medications. The consensus shows that current treatments, including the use of antibiotics such as doxycycline, cefuroxime, and others, are the most clear-cut choices. However, with PTLDS affecting up to 20% of those with Lyme disease, more options are needed to prevent Lyme disease or treat PTLDS to help patients. The most promising options include actively developing vaccinations such as VLA15 and antibiotics such as hygromycin A and azlocillin, which are being investigated for safety and efficacy. Moreover, each of these options allows researchers to explore better options for Lyme disease treatment and also more insight into the true root cause of PTLDS. The current literature suggests the prevention of Lyme disease symptoms and infection to prevent PTLDS, and more research and investigation are required to find direct treatments and causes for PTLDS. Currently, no treatment option exists that will directly treat PTLDS, and the first step appears to be discovering the pathology of the disease. While certain theories exist, the leading hypotheses pertain to lingering or drug-resistant *B. burgdorferi*, autoimmune attacks, remaining peptidoglycans, and central sensitization. Despite the array of theories, more in-depth research is certainly required in each area to help find a suitable treatment for PTLDS.

Additionally, the creation of Lyme disease-specific treatments and procedures is in the works. Still, more research directed to this field is essential to combat the growing number of cases and the severity of PTLDS or any other lingering symptoms. Currently, studies on PTLDS are lacking and more research is required to meet the growing demand for treatment. Lyme disease, which was primarily centralized in the northeast on the coast, has expanded to affect more inland areas, including expansion into the Midwest. Current treatment includes antibiotics such as doxycycline, cefuroxime, and amoxicillin, as well as any relevant supportive treatment. Goals of developing treatment hope to prevent or treat Lyme disease effectively to stop the development of PTLDS. Current PTLDS treatment is mostly supportive with a need for more research into direct treatments. New ongoing developments include the VLA15 vaccine and new antibiotics such as hygromycin A and azlocillin.
